# Ranking Self-reported Gastrointestinal Side Effects of Pharmacotherapy in Sarcoidosis

**DOI:** 10.1007/s00408-020-00323-8

**Published:** 2020-01-20

**Authors:** M. Drent, V. L. J. Proesmans, M. D. P. Elfferich, N. T. Jessurun, S. M. G. de Jong, N. M. Ebner, E. D. O. Lewis, A. Bast

**Affiliations:** 1 Department of Pharmacology and Toxicology, FHML, Maastricht University, Maastricht, The Netherlands; 2 Department of Pulmonology, ILD Center of Excellence, St. Antonius Hospital, Koekoekslaan 1, 3435 CM Nieuwegein, The Netherlands; 3 ild care foundation Research Team, Ede, The Netherlands; 4 Venlo Campus, Maastricht University, Venlo, The Netherlands; 5 Netherlands’ Pharmacovigilance Centre Lareb, ’s Hertogenbosch, The Netherlands

**Keywords:** Gastrointestinal side effects, Glucocorticoids, Methotrexate, Pharmacotherapy, Sarcoidosis, Side effects, Treatment

## Abstract

**Background:**

Clinical manifestations of sarcoidosis vary widely, depending on the intensity of the inflammation and the organ systems affected. So far, no curative treatment exists; the disease can only be suppressed. All treatment options cause side effects affecting quality of life. The aim of this study was to establish and rank the prevalence of self-reported gastrointestinal side effects of drugs used in the treatment of sarcoidosis.

**Methods:**

A cross-sectional web-based anonymous survey about complaints and side effects was conducted among sarcoidosis patients in the Netherlands, United Kingdom, and United States of America.

**Results:**

Of the participants, 70% were being treated with one or more drugs. The most important reported side effect was weight gain, associated with increased appetite among prednisone users (as monotherapy as well as in combination with other drugs). Methotrexate (MTX) users especially experienced nausea, with monotherapy as well as combination therapy. Vomiting and weight loss were most prominent among azathioprine and mycophenolate mofetil (MMF) users, whereas diarrhoea was frequently mentioned by MMF and MTX users. The reported side effects of hydroxychloroquine were generally rather mild.

**Conclusion:**

The current study ranked the gastrointestinal side effects associated with pharmacotherapy in sarcoidosis patients. Pharmacotherapy does have multiple gastrointestinal side effects. The strongest association between a reported side effect and drug use was that of weight gain associated with increased appetite among prednisone users. It would therefore be useful for future research to look further into dietary interventions to counter these side effects and reduce their burden.

## Introduction

Sarcoidosis is a multisystem inflammatory disorder of unknownetiology. The disease is characterized by the formation of non-caseating granulomas in various organ systems, mainly the lungs and lymphatic system, but any organ can be involved [1]. The exact cause of sarcoidosis is still unknown, but is likely to depend on both genetic and environmental factors, probably antigen-driven. The natural history and prognosis of sarcoidosis are highly variable. Within 3 years of diagnosis, over 50% of patients have achieved remission, while within a decade approximately one-third of patients have persistent disease, leading to a significant burden on their lives [1, 2]. The management of sarcoidosis is challenging due to the heterogeneity of its organ manifestations and its clinical course, as well as the potential side effects of current immunosuppressive therapy. The lack of approved drugs tested in randomized controlled trials hampers the development of standardized treatment protocols for sarcoidosis [1]. As a general rule, deterioration that threatens any organ function warrants treatment. Systemic sarcoidosis treatment should generally be given to prevent end-organ damage.

There are three lines of therapy for sarcoidosis. Oral glucocorticoids (GCs) are the initial first-line therapy for symptomatic patients. Non-specific immunosuppression with prednisone remains the first-choice systemic therapeutic option [1]. Second-line agents in sarcoidosis include MTX, azathioprine (AZA), leflunomide, and hydroxychloroquine (HCQ), although the available evidence supporting their use, like that of prednisone, is limited. Of the second-line agents for sarcoidosis, MTX has been the most widely studied and guidelines have been established [3]. MTX has been demonstrated to be effective and less toxic than AZA [3, 4]. Leflunomide is similar to MTX in action but with a different toxicity profile [5–7]. It is associated with less nausea and pulmonary toxicity [8], but it can cause peripheral neuropathy. More recently, mycophenolate mofetil (MMF) was reported to be useful when other drugs had to be discontinued due to toxicity; however, evidence for the effectiveness of this product is rather limited [9]. It was suggested to be successful in neurosarcoidosis and cutaneous sarcoidosis [10]. HCQ and chloroquine are antimalarial agents that have proved useful to treat skin manifestations [11] and abnormalities of calcium metabolism [12]. Third-line treatment consists of biologicals (tumor necrosis factor-alpha (TNF-α) inhibitors), and is currently reserved for patients non-responsive to first- or second-line treatment. Guidelines have been established to help identify which patients to treat, and include dosing and monitoring schemes [13]. Infliximab is the most widely studied and administered monoclonal antibody for the treatment of various manifestations of severe sarcoidosis [13]. Adalimumab has also been used, although to a lesser extent [13, 14]. Nowadays, biosimilars are commonly used and have proved to be effective and safe as well [15–17].

The drugs currently used to treat various manifestations of sarcoidosis tend to cause side effects, including gastrointestinal side effects, which then further increase the burden of this disease [1]. Since the importance of patient participation in healthcare decisions has been increasingly acknowledged [18], and studies from a patient perspective are important [19], we conducted an online survey of self-reported gastrointestinal side effects.

The aim of this study was to establish and rank the prevalence of self-reported gastrointestinal side effects of drugs used in the treatment of sarcoidosis.

## Methods

### Study Design

In cooperation with the Dutch Sarcoidosis Patient Society (Sarcoidose.nl), the ild care foundation designed a questionnaire about side effects of drugs used to treat sarcoidosis-related problems. The questionnaire was used in a cross-sectional web-based anonymous survey conducted from June 2018 to December 2018 among a sample of sarcoidosis patients in the Netherlands, United Kingdom, and United States. This study was performed in accordance with the Declaration of Helsinki and its amendments.

### Study Subjects and Procedure

Patients were recruited through membership of the Dutch Sarcoidosis Society, Sarcoidose.nl, via the society’s newsletter and through an advertisement at the ILD Center of Excellence at Nieuwegein in the Netherlands, as well as through membership of SILA (Sarcoidosis and Interstitial Lung Association) in the UK and FSR (Stop Sarcoidosis Research Foundation) in the USA. Participating patients were proficient in Dutch or English and had internet access. Patients were enrolled in NL, UK, and USA without incentives, since the survey was anonymous. The survey was developed using the online questionnaire tool *Surveymonkey* (www.surveymonkey.com). The questions concerned demographics (gender, age, duration of sarcoidosis), the burden of sarcoidosis and symptoms experienced by the patients, the use of medication, and side effects. Respondents were asked to complete the questionnaire even if they had never experienced any problems with their drugs. Those patients who reported not using any drugs to treat sarcoidosis were regarded as controls.

### Statistical Analysis

All statistical analyses were performed using SPSS statistical software (22.0 for Windows; SPSS Inc., IL, USA). The adverse side effects of GCs, MTX, TNF-α inhibitors, AZA, HCQ, and mycophenolate mofetil (MMF) were tested by assessing the variables nausea, vomiting, diarrhoea, weight loss, weight gain, appetite loss, loss of taste perception, loss of smell perception, and dry mouth, using a logistic regression analysis with a logit link. In this logistic regression, the side effects were used as response variables, while the various drugs were used as explanatory variables. The data were analyzed and summarized in a forest plot developed using Metaexcel, an add-on for Excel.

## Results

Table 1 summarizes the demographic and clinical data of our sarcoidosis sample. In total, 937 patients completed the questionnaire: 282 patients were using no drugs, 416 used prednisone either alone or in combination, 272 were using MTX, 128 TNF-α inhibitors, 53 AZA, 107 HCQ, and 29 MMF.

**Table 1:** Summary of the demographic and clinical data of the three sarcoidosis samples

	The Netherlands	United Kingdom	United States	Total
Number	646	37	254	937
Age, mean ± SD	54 ± 11	53 ± 10	55 ± 10	55 ± 11
Male (%)	38.2	18.9*	30.0	35.3
Time since diagnosis (%)				
< 1 year	11.8	13.5	4.0*	9.8
1–2 year	12.9	21.6	10.8	12.7
2–5 year	23.8*	35.1	32.7	26.6
> 5 year	51.5	29.7*	52.6	50.9
BMI (kg/m^2^), mean ± SD	28.7 ± 5.8	28.7 ± 6.1	31.5 ± 7.9*	29.4 ± 6.5
Smoker (%)	4.5	11.1	5.3	5.0
Non-smoker (%)	36.8	47.2*	29.9	35.4
Former smoker (%)	58.7	41.7	64.8	59.6
Non-drug users (%)	33.1	32.4	22.0*	30
Drug users (%)				
Prednisone	40.4*	59.5	52.4	44.4
Methotrexate	28.9	16.2	31.1	29.0
TNF-α inhibitors	13.2	2.7	16.5	13.7
Azathioprine	4.3	5.4	9.1*	5.7
Hydroxychloroquine	9.1	10.8	17.3*	11.4
Mycophenolate mofetil	2.0	0	6.3*	3.1
Duration of use of current drug (%)				
< 6 months	16.2	16.0	12.5	15.2
6–12 months	11.7	12.0	11.1	11.6
> 1 year	72.0	72.0	76.4	73.3

Data are expressed as mean ± SD or percentage if appropriate

*BMI* body mass index

^*^Value differs significantly from the two other countries (*p* < 0.05)

Table 2 presents the numbers of participants using a single drug or a combination of two drugs. Sixty patients in our sarcoidosis sample were being treated with more than two drugs, and were not included in Table 2, while 282 (30%) of our sample were currently not being treated for their sarcoidosis with any of the above-mentioned drugs. Of the drug users (*n* = 655, 70%), 380 (58%) were using one drug and 214 (33%) were using two drugs (see Table 2). Forty-nine (7%) patients were being treated with combinations of three drugs and 11 (2%) with four or five drugs. The combination of prednisone with MTX was most common (88 patients out of those using two drugs, 32 out of those using three drugs [15 combined with TNF-α-inhibitors, 3 combined with AZA and 14 with HCQ], and 9 patients out of those using four drugs).

**Table 2:** Number of sarcoidosis patients studied using a single drug or a combination of two drugs, subdivided into glucocorticoids (GCs) like prednisone, methotrexate (MTX), TNF-alpha inhibitors (TNF-α-i), azathioprine (AZA), hydroxychloroquine (HCQ), and mycophenolate mofetil (MMF)

	GC	MTX	TNF-α-i	AZA	HCQ	MMF
GC	206	88	21	18	18	10
MTX	88	89	38	0	13	0
TNF-α-i	21	38	26	3	2	3
AZA	18	0	3	13	2	0
HCQ	18	13	2	2	39	0
MMF	10	0	3	0	0	7

**Fig. 1:**
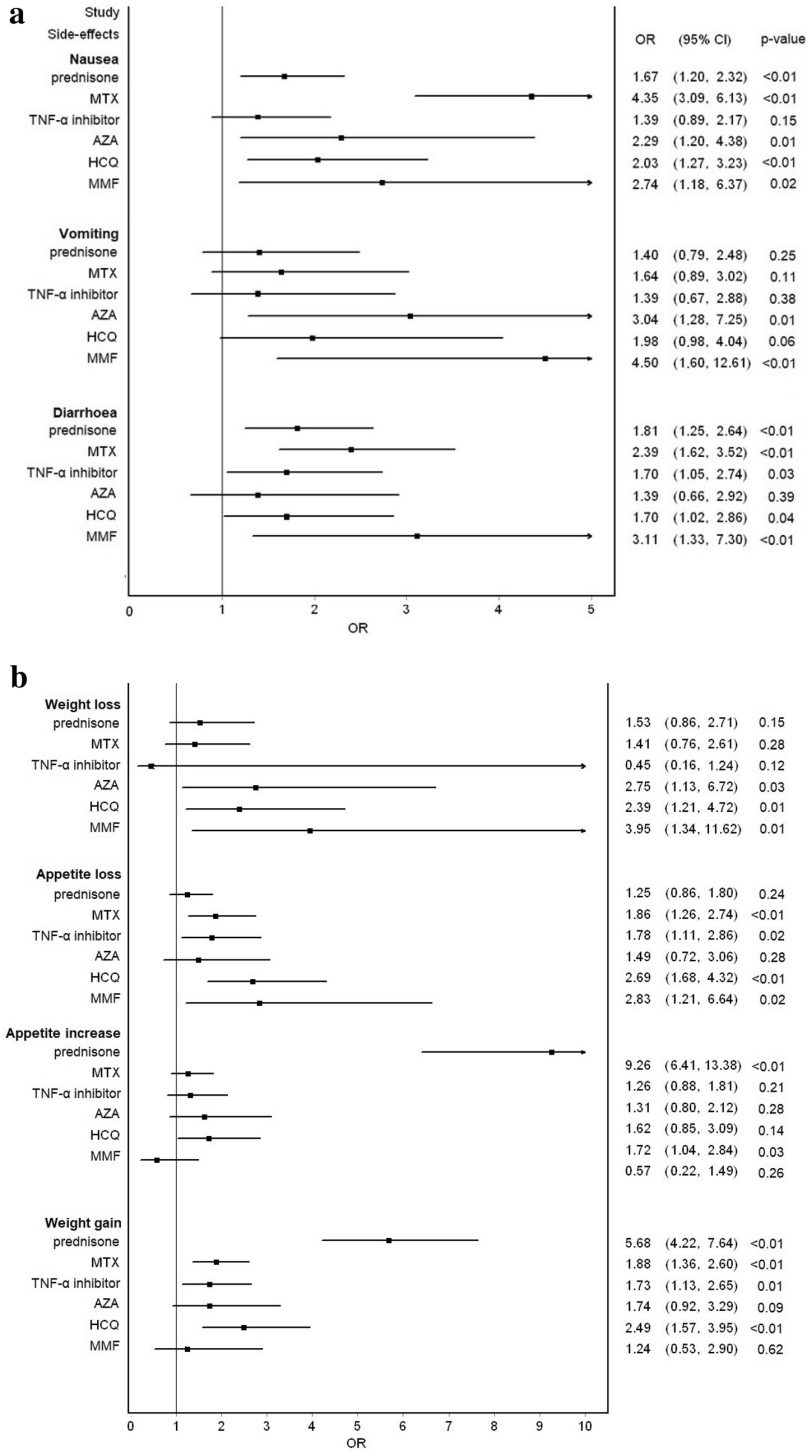
**a** Forest plot of gastrointestinal side effects (nausea, vomiting, and diarrhoea), ranked for the various drugs used in the treatment of sarcoidosis, in comparison to non-drug users. **b** Forest plot of gastrointestinal side effects (weight loss, appetite loss, appetite increase, and weight gain) ranked for the different drugs used in the treatment of sarcoidosis, in comparison to non-drug users

Two forest plots summarize the occurrence of gastrointestinal side effects, ranked for the different drugs used for the treatment of sarcoidosis (Fig. 1a, b). Prednisone use was associated with increased appetite (OR 9.26) and with weight gain (OR 5.68). This effect was also evident for those using combinations with the other drugs (data not shown).

MTX users reported suffering most from nausea (OR 4.35). This effect occurred both with monotherapy and with combinations with other drugs (data not shown). All other drugs except TNF-α-inhibitors also had nausea as a side-effect, although to a lesser extent. AZA and MMF were especially associated with vomiting (OR 3.04 and 4.50, respectively). MMF use contributed most to diarrhoea complaints (OR 3.11). AZA, HCQ, and MMF users showed significantly more weight loss, with odds ratios ranging from 2.39 to 3.95.

Weight gain was associated with increased appetite (*r* = 0.489, *p* < 0.001), while weight loss was associated with loss of appetite (*r* = 0.468), as well as nausea (*r* = 0.193), vomiting (*r* = 0.184), and diarrhoea (*r* = 0.133; all *p*’s < 0.001).

**Fig. 2:**
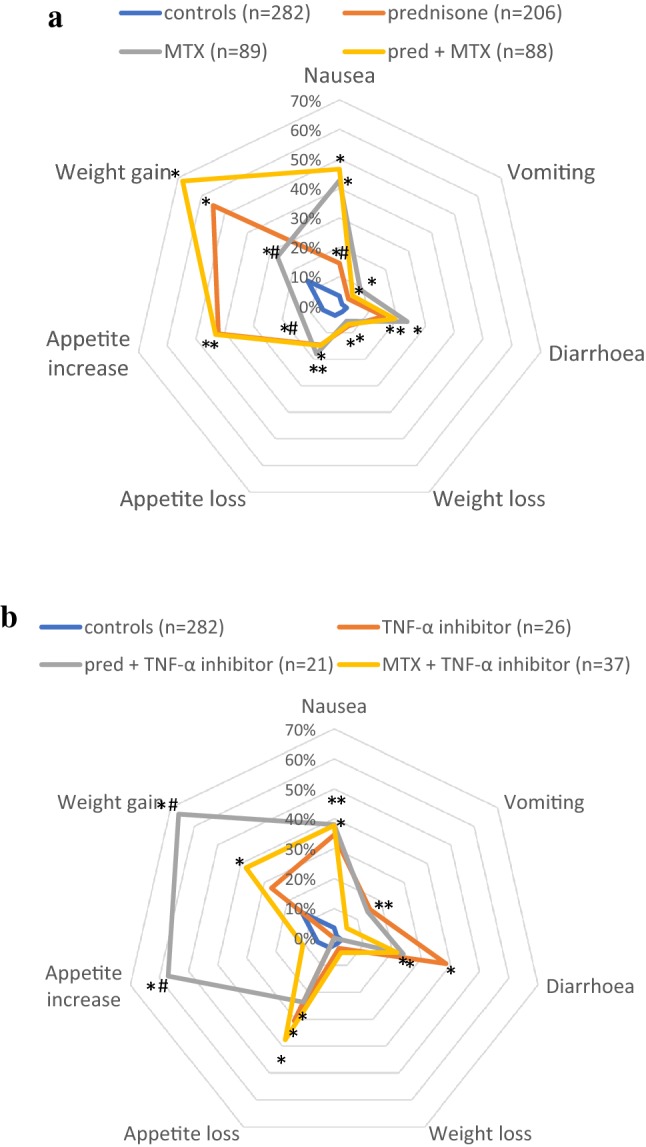
**a** Spider plot of gastrointestinal side effects of prednisone, methotrexate (MTX), and the combination of prednisone and MTX therapy in sarcoidosis. *Group differs significantly (*p* < 0.05) from the control group (non-drug users). ^#^Group differs significantly from the other drug groups (*p* < 0.05). **b**. Spider plot of gastrointestinal side effects of TNF-α inhibitors: monotherapy or in combination with prednisone or methotrexate (MTX). *Group differs significantly (*p* < 0.05) from the control group (non-drug users). ^#^Group differs significantly from the other drug groups (*p* < 0.05)

The spider plots (Fig. 2a, b) show significantly more appetite loss, nausea, and diarrhoea complaints among the treatment groups compared to the controls (*p* < 0.05). Vomiting also occurred significantly more among the different treatment groups, except for prednisone monotherapy. The same goes for the weight gain complaints, which increased in all treatment groups except the TNF-α-inhibitor monotherapy (*p* < 0.05).

Comparing the different drug groups, Fig. 2a shows that prednisone monotherapy as well as prednisone combined with MTX increased appetite and weight gain complaints compared to MTX monotherapy (*p* < 0.05). As regards nausea, MTX monotherapy and combination therapy with prednisone caused a significantly higher burden than prednisone monotherapy.

Among the different TNF-α inhibitor treatment options (Fig. 2b), the combination of prednisone and TNF-α inhibitors significantly increased appetite and weight gain complaints (*p* < 0.05). Diarrhoea complaints were also higher in the TNF-α inhibitor group, but the difference was not significant (*p* = 0.05).

Duration of illness, age, and gender mainly had effects on the prescription of drugs. No important relationships were found between these parameters and the side effects. A more detailed evaluation of the effect of prednisone on weight and appetite showed that these effects were smaller in men than in women, and that age was negatively correlated with the magnitude of the effect, but that the duration of the disease did not have an influence. As regards the observed effect of MTX on nausea, gender did not play a role in this, nor did the disease duration affect the nausea effect elicited by MTX. Age was negatively correlated with the nausea effect of MTX, as younger patients experienced more nausea as a side effect.

## Discussion

This study has assessed the associations between drugs and side effects of pharmacotherapy among 932 sarcoidosis patients, and ranked their self-reported gastrointestinal side effects. The most frequently reported side effect was weight gain, associated with increased appetite, among prednisone users. Nausea was experienced by MTX users (as monotherapy as well as in combination with other drugs). Both vomiting and weight loss were most prominent in AZA and MMF users, whereas diarrhoea was mainly reported by MMF and MTX users. The reported side effects of HCQ were generally rather mild.

Sarcoidosis is characterized by inflammation, and current treatment mainly consists of anti-inflammatory drugs. Since the cause of the disease is still unknown, it is important to carefully evaluate the treatment of choice, considering its possible benefits and drawbacks. In line with previous studies, the most important side effect related to prednisone use in this study was weight gain and increased appetite [20–22]. These effects were smaller in men than in women. This is probably due to a difference in BMI. This is problematic because obesity is an increasing health problem, also for patients with sarcoidosis [23, 24]. Apart from prednisone, all other drugs ranked in this study did not result in substantial weight gain. Moreover, prolonged prednisone therapy is associated with other significant side effects as well, such as diabetes, infections, fluid retention, muscle weakness, glaucoma, cataracts, insomnia, mood swings, personality changes, osteoporosis, and osteonecrosis of the femoral head [21]. The risk of side effects appears to depend on both the cumulative dose and the duration of prednisone use [21], whereas the efficacy of prednisone was not found to be related to the dosage [25]. Given the severe adverse events accompanying long-term treatment with prednisone, timely use (before an unacceptable increase in BMI) of an appropriate cytotoxic agent with steroid-sparing potency is to be recommended. In case of an increased BMI it might even be useful to start straight away with a GC-sparing agent. Another approach might be to consume appropriate flavonoid-rich food in conjunction with corticosteroids, which has appeared to increase the efficacy of corticosteroids, thereby reducing the dose required for anti-inflammatory action, with less risk of developing side effects [26].

The existence of oxidative stress in sarcoidosis patients is evident from the decrease in overall plasma antioxidant values. Clearly, antioxidant supplementation, such as quercetine, might be helpful to restore the antioxidant levels in these patients [27–29]. It has even been suggested that the anti-inflammatory effect of flavonoids might result from their maintaining effect on the ability of cortisol to cope with pro-inflammatory triggers and the prevention of overreactive inflammatory processes [26].

Generally, MTX and AZA are regarded as the second-line treatments, with MTX being preferred to AZA because of lower rates of discontinuation [14]. In terms of gastrointestinal side effects, MTX was associated with increased nausea, diarrhoea, decreased appetite, weight gain, and dry mouth (*p* < 0.05). The significantly increased nausea but not vomiting is in line with previous data, as is the occurrence of diarrhoea [4]. Weight gain complaints were also found in studies of rheumatoid arthritis (RA) patients who used MTX [30]. The OR for weight gain was 1.88 among MTX users, which is significantly lower than the OR for weight gain among prednisone users (5.68). Hence, MTX could reduce weight gain when used in conjunction with prednisone as a corticosteroid sparing drug, as reported in a previous study [31]. AZA was associated with nausea and vomiting, which is in line with previous research [32].

TNF-α inhibitors are regarded as third-line treatment. The current study showed that TNF-α inhibitor use was associated with decreased appetite, weight gain, and diarrhoea (with or without concurrent drug use). However, the largest study using infliximab in sarcoidosis, by Baughman et al., reported no gastrointestinal side effects [33]. Meanwhile, gastrointestinal adverse drug reactions associated with the use of infliximab have been recognized and even included in the summary of product characteristics of Remicade® [34]. Sfriso et al. reported weight gain in patients with RA under treatment with TNF-α inhibitors [35]. A previous study among patients at a rheumatology clinic also reported weight gain in patients with various indications treated with TNF-α inhibitors [36]. However, no significant changes in appetite were noted among this group [36].

HCQ use was associated with nausea, diarrhoea, decreased appetite, and weight loss in some cases, but increased appetite and weight gain in others. Weight gain among HCQ users has been suggested to be associated with a reduction of gut microbiota caused by the drug [37]. Mycophenolate mofetil was associated with nausea, vomiting, and diarrhoea, which was also found in previous studies [9, 10]. Weight loss, decreased appetite, and loss of taste perception were also noted among the patients in the present study.

Although drug-induced taste disturbances are common, they have failed to attract the attention of most clinicians. Similarly, taste disorders have been largely ignored by pharmaceutical companies during preclinical drug development [38]. Only the summary of product characteristics of MTX tablets mentions taste disorders as a possible adverse drug reaction. Recently, Schiffman published a review about the influence of medications on taste and smell [39]. However, the incidence and prevalence of medication-induced chemosensory disorders referred to in this review related to the use of immunosuppressant drugs, and to other drugs used to treat sarcoidosis are lacking. In the present study the influence of all reported drugs on taste and smell perception appeared to be rather mild and minor compared to the other gastrointestinal side effects. This was in accordance with previous studies, which reported a possible effect of corticosteroids on taste perception [40]. Dry mouth was most prominent in prednisone users, whereas loss of taste perception was most prominent in MMF users.

The large patient group included in our study enabled us to attribute a clear statistical significance (*p*-value) to the actual contribution of the side effects induced by drug treatment, as indicated in the Forest plot of Fig. 1. Moreover, the results are consistent with those of other studies which suggest that many of the problems encountered are related to the medications rather than the sarcoidosis.

### Limitations

One of the limitations of this study is that information about disease severity was lacking, so the impact of disease severity on the reported side effects could not be established. Another limitation is that the symptoms were self-reported and not verified by a health care professional. However, this is what patients experience and associate with the drugs used for their sarcoidosis.

### Recommendations

The data from our study show that all drugs reported on cause some burden of gastrointestinal side effects. Therefore, it would be useful to look into possible dietary interventions to minimize this burden, as well as the use of other drugs to counter the side effects. Overweight and obese patients should avoid GC and should be considered for second-line treatment directly. Strategies to manage gastrointestinal side effects caused by one of the sarcoidosis drugs start with the advice to take the tablets during a meal, not on an empty stomach, which might reduce the peak dose. In the case of MTX-induced gastrointestinal side effects, including mucositis, adding folic acid to the diet as well as splitting the oral dose should be considered, provided the total MTX dose is ingested within a 12-h period. Parenteral administration or an alternative immunosuppressive drug should be considered in case of persistent intolerance [3]. In case of incessant diarrhoea, the next step may be rehydration and anti-diarrhoeal medication, for example loperamide, and in case of constant nausea, anti-emetics. Furthermore, antacids are recommended for indigestion [41]. Although there is no ‘one-size-fits-all’ policy for persistent side effects, supervised dose reduction and re-titration may be required once the symptoms have subsided.

Our findings can be used to support patients and their care providers in the choice of drugs with regard to gastrointestinal side effects. With previous research findings already pointing at the possible role of food components and additives in inflammatory lung diseases [27–29], it could be interesting to explore the possibilities of implementing dietary interventions in sarcoidosis treatment in order to reduce the side effects or counter the disease.

## Conclusion

The current study ranked the gastrointestinal side effects associated with pharmacotherapy in sarcoidosis patients, in terms of frequency. The currently used pharmacotherapy causes multiple gastrointestinal side effects. The most important reported side effect was weight gain associated with increased appetite among prednisone users (both as monotherapy and in combination with other drugs). Since obesity is still an increasing health problem, alternatives that can be used as first-line treatment in sarcoidosis are urgently needed. The next most important side effect appeared to be nausea, experienced especially by MTX users (as monotherapy as well as in combination with other drugs). It would therefore be useful for future research to look further into dietary interventions to counter these side effects and reduce their burden.

## Data Availability

The datasets used and/or analyzed during the current study are available from the corresponding author on reasonable request.
